# Application of “Systems Vaccinology” to Evaluate Inflammation and Reactogenicity of Adjuvanted Preventative Vaccines

**DOI:** 10.1155/2015/909406

**Published:** 2015-08-25

**Authors:** David J. M. Lewis, Mark P. Lythgoe

**Affiliations:** ^1^Clinical Research Centre, University of Surrey, Guildford GU2 7AX, UK; ^2^Clinical Research Facility, Imperial College Healthcare NHS Trust, London W12 0HS, UK

## Abstract

Advances in “omics” technology (transcriptomics, proteomics, metabolomics, genomics/epigenomics, etc.) allied with statistical and bioinformatics tools are providing insights into basic mechanisms of vaccine and adjuvant efficacy or inflammation/reactogenicity. Predictive biomarkers of relatively frequent inflammatory reactogenicity may be identified in systems vaccinology studies involving tens or hundreds of participants and used to screen new vaccines and adjuvants in *in vitro*, *ex vivo*, animal, or human models. The identification of rare events (such as those observed with initial rotavirus vaccine or suspected autoimmune complications) will require interrogation of large data sets and population-based research before application of systems vaccinology. The Innovative Medicine Initiative funded public-private project BIOVACSAFE is an initial attempt to systematically identify biomarkers of relatively common inflammatory events after adjuvanted immunization using human, animal, and population-based models. Discriminatory profiles or biomarkers are being identified, which require validation in large trials involving thousands of participants before they can be generalized. Ultimately, it is to be hoped that the knowledge gained from such initiatives will provide tools to the industry, academia, and regulators to select optimal noninflammatory but immunogenic and effective vaccine adjuvant combinations, thereby shortening product development cycles and identifying unsuitable vaccine candidates that would fail in expensive late stage development or postmarketing.

## 1. Introduction

As preventive vaccines are typically administered to healthy people including infants, children, and persons with comorbidities, there is particular emphasis on safety, with an expectation of high benefit-risk ratio. This is especially the case with vaccines administered during pregnancy, where enhanced regulatory concerns have been expressed [1]. Modern vaccines therefore frequently make use of recombinant technology to manufacture purified pathogen subunit molecules, an approach that has allowed rational vaccine design, cGMP manufacture, and reproducibility of immune responses and acceptable adverse events profiles. A significant benefit of recombinant subunit vaccines, especially bacterial, is that the exclusion of nonantigenically relevant pathogen cellular components with inherent immunostimulatory properties greatly reduces activation of innate immune pathways and consequent inflammation, thereby reducing undesirable reactogenicity. A downside of this minimalist approach, seen, for example, with the switch from whole cell to acellular subunit pertussis vaccines, is that it may lead to reduced immunogenicity of the vaccine and coadministered vaccines, especially at the extremes of age. Therefore, to preserve efficacy without reactogenicity, various selective vaccine adjuvants have been developed with the aim of restoring immune activation to retain potency, but without inducing unacceptable inflammation. While the mode of action of adjuvants is diverse and remains unknown for even some licensed adjuvants, many typically harness components of the innate immune system [2–4], such as pattern recognition receptors (PRR), that detect infection by mimicking toll like receptor (TLR) agonists [5]. While immunostimulatory adjuvants offer the potential to beneficially modulate the immune response to antigens, their use may raise safety concerns amongst the general public and regulators, related to speculation that they may induce overproduction of inflammatory and pyrogenic molecules [6], especially in outbred human populations where genetic or environmental factors may amplify or modify innate and adaptive immunity [7] in potentially unpredictable ways.

Furthermore, while rodent species are often employed in basic immunology research and preclinical toxicology testing, marked differences between murine and human innate and adaptive immunity exist [8, 9]. This is in part due to species-specific differences in receptor responses and the tissue distribution of innate system molecules and pathways [10]. The situation may be further compounded for some species such as ferrets, used in models of influenza infection and immunization, where suitable immunology reagents may be totally unavailable. It is therefore increasingly acknowledged that traditional toxicological approaches to evaluate adjuvanted vaccines in preclinical models [8, 11] may fail to identify an increased risk of adverse events following immunization (AEFIs) [12], which may emerge in clinical development [13] or even postmarketing [14]. Additionally, given the wide variety of vaccine adjuvants and their targets or modes of action and combinations with different antigens in a variety of formulations that may modulate the adjuvant effect, predictive immune parameters and toxicology readouts from preclinical studies for a particular adjuvanted vaccine may not be predictive across animal species or different adjuvanted vaccines.

While the occurrence of relatively frequent immediate inflammatory reactogenicity such as fever and injection site reactions may be possible to model in preclinical models, the risks of rare but serious AEs associated with vaccines that could occur in certain at-risk populations (as seen with initial introduction of rotavirus vaccine [15]) are unlikely to be revealed in preclinical studies or during clinical development and may require long follow-up of subjects in clinical trials. Recent late stage clinical trials [16] which have shown either a complete or a partial lack of efficacy or raised safety concerns, despite encouraging preclinical data, have reinforced the need for reliable predictive biomarkers of safety and efficacy that could be used in preclinical studies to prioritize available candidates and in early clinical development to avoid failure during lengthy and costly Phase 2b/3 clinical trials.

There is therefore an urgent unmet need to develop new technologies to identify novel biomarkers of adjuvant toxicity, especially experimental medicine models harnessing advances in human immunology, for subsequent validation in clinical trials, which will enhance preclinical and clinical safety evaluation of products containing existing and novel vaccine adjuvants.

## 2. Systems Vaccinology: Biomarkers of Vaccine Safety and Efficacy

A biomarker is a characteristic measured objectively at a single time point and evaluated as an indicator of a physiological or pathological process or pharmacological response(s) to a therapeutic intervention such as vaccine-induced protective immunity [12]. Several published studies have used vaccines in translational studies in which multiparametric technologies such as transcriptomics, metabolomics, and proteomics are used to dissect out fundamental mechanisms of reactogenicity and efficacy in human and animal models, in which whole blood or separated cell population gene expression, cytokine responses, and cellular and humoral immune responses are integrated [17, 18]. One benefit of the systems approach is that observations from small number of clinical samples can be further explored in animal models [19]. A systems-wide analysis has been used to identify novel mechanisms regulating vaccine responses [20, 21], now often referred to as “systems vaccinology” [22]. While a number of publications have demonstrated underlying mechanisms possibly associated with vaccine efficacy (as measured by immunogenicity), there are often conflicting outcomes due to a lack of standardization of systems biology techniques and bioinformatics analyses [23, 24]. Furthermore, different vaccine antigens or adjuvant systems are likely to induce different innate and adaptive responses, making extrapolation from different trials challenging.

While a number of research projects and consortia have been initiated to identify biomarkers of vaccine* efficacy* (such as the European Commission-Funded High Impact Project ADITEC, http://www.aditecproject.eu/ [25]), much less has been carried out in the area of vaccine* safety* and* reactogenicity*. Wang et al. indirectly observed that upregulation of genes associated with innate immunity, cytokine production, and responses to virus infection, particularly IFN-inducible genes, observed in nonhuman primates did correlate with adverse events seen in human trials [26]. An adverse reaction not uncommonly seen after immunization is fever. Activation of innate immunity and inflammation induces a febrile response [27], probably via the action of pyrogenic cytokines such as IL-1*β*, IL-6, and TNF-*α* [28] or prostaglandins such as PGE2 synthesized by liver Kupffer cells. Despite being normally an unwelcome but tolerable reaction to immunization, the association of febrile convulsions in infants with some vaccines such as whole cell pertussis is a safety concern. As a result, attempts to model the cytokine and prostaglandin profile after immunization in preclinical toxicology assays employing rabbits and human cell lines have been undertaken with some success [29]. Indeed, a systems approach was successfully applied to retrospectively identify underlying factors (i.e., biomarkers) responsible for the unexpected increase in febrile seizures in children associated with a specific trivalent influenza vaccine, by combining human, animal, cell line, and primary cell culture experiments with gene profiles and cytokine readouts [30]. This opens the possibility to screen vaccine antigen combinations or production methods for reactogenicity before release, using systems vaccinology. A recent meeting on the use of biomarkers for assessment of vaccine safety concluded that while the integration of high throughput multiparametric data from* in vitro*, preclinical, and clinical evaluations of vaccines and adjuvants in systems analyses was a powerful tool to identify basic mechanisms involved in vaccine and adjuvant reactogenicity and efficacy, considerable effort is still required to simplify, harmonize, and standardize these approaches if the data are to be practically applicable to vaccine and adjuvant development and safety monitoring [31].

## 3. BIOVACSAFE: Biomarkers of Vaccine Immunosafety

**Figure 1:**
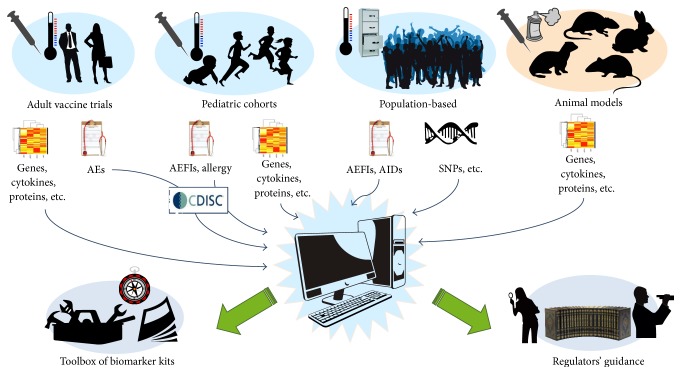
Integration of multiparametric “omics” data with clinical events from human clinical trials, together with population genetics, pediatric cohorts, and animal models in the BIOVACSAFE project.

In 2011, the 5-year BIOVACSAFE project initiated, for the first time, a program of activities that integrate a systems biology approach with animal models and established clinical evaluation of reactogenicity after immunization or natural infection, and population-based genetics, to identify biomarkers of vaccine safety and reactogenicity (see Figure 1). The 30M€ project, coordinated by the University of Surrey, UK, and Novartis Vaccines, Italy, and funded by the Innovative Medicine Initiative, is a unique public-private partnership involving four EFPIA member pharmaceutical companies (GSK Bio, Sanofi Pasteur, Novartis Vaccines, and deCODE, AmGen) with 17 academic organizations, SMEs, and public institutions. Organized into work packages, it is the first truly systematic approach to apply systems vaccinology to vaccine and adjuvant safety rather than efficacy.

### 3.1. Human Experimental Medicine Studies of Systems Vaccinology

**Figure 2:**
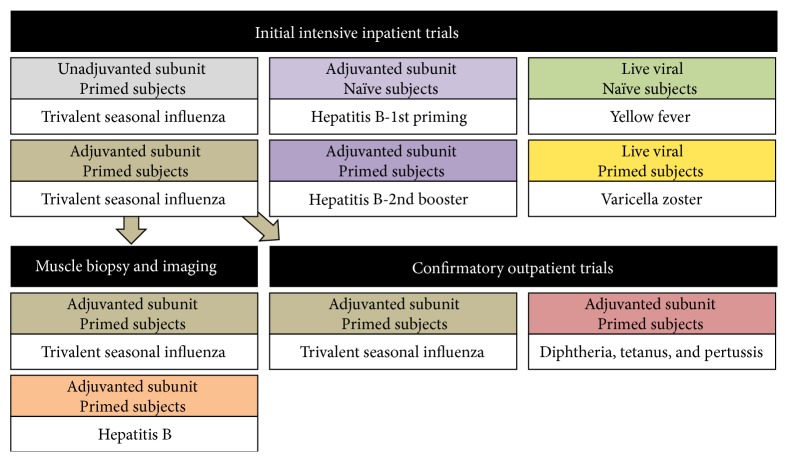
Organization and sequence of head-to-head comparison of adjuvanted and unadjuvanted vaccines, with live vaccines in experimental medicine studies within the BIOVACSAFE project.

The BIOVACSAFE project has significantly refined the previous approach of systems vaccinology in which a “training set” of data are generated from small clinical studies with around 15 healthy adult subjects per vaccine group, to identify putative correlations or biomarkers associated with a desired outcome (such as immunogenicity) which are then confirmed in larger “confirmatory studies” typically involving over 100 participants. For example, unlike previous efforts in which different vaccines are evaluated in separate trials (in which many variables may be different such as population, environment, and methodology), BIOVACSAFE has taken a highly structured approach to perform head-to-head comparisons in naive or immune populations using prototypic vaccines and adjuvants (see Figure 2) performed as separate groups within the same protocol at the same clinical site. Both adjuvanted (MF59C, AS04C, and alum) and unadjuvanted vaccines are compared head-to-head in primed (influenza and booster immunization with hepatitis B vaccines) and naive subjects (priming immunization with hepatitis B vaccine). Alongside these subunit adjuvanted vaccines, live viral vaccines were tested in naive (yellow fever) and immune (varicella) populations. This structure is unique to BIOVACSAFE and will allow biomarkers unique to each vaccine-target combination to be discriminated from more generalized biomarkers common to a number of vaccines or target populations.

**Figure 3:**
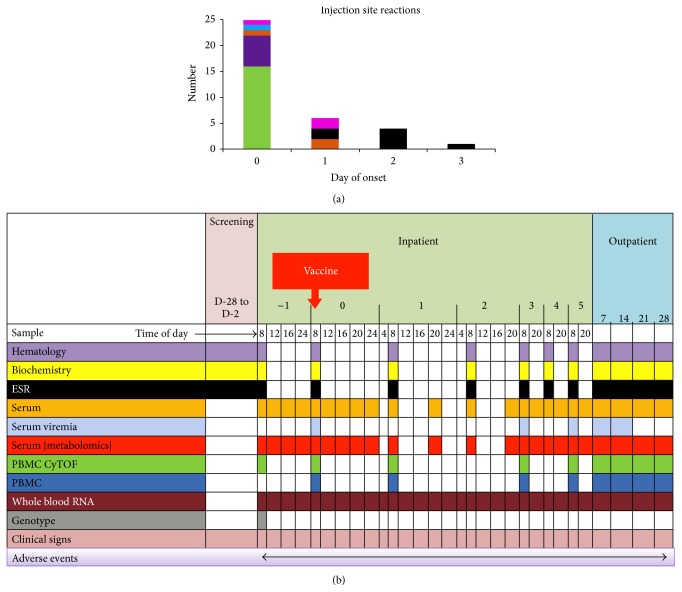
(a) Day of onset for injection site reaction for five different vaccines plus saline placebo (blue), (b) schedule of sampling for systems vaccinology parameters during intensive inpatient studies.

Furthermore, in the typical systems vaccinology scenario, clinical samples are taken on days 0, 3, 5, and 7 and at weekly intervals thereafter to characterize immune responses in an outpatient setting, with the schedule being highly influenced by convenience of study organization, which misses very early time points when innate immune cells may be most active in setting the direction of subsequent immune response and reactogenicity. In contrast, the BIOVACSAFE clinical “training trials” were conducted in an* inpatient setting* in which diet, exercise, sleep, alcohol, and tobacco were strictly regulated to ensure minimal background variability that could interfere with subtle physiological events after immunization. This allowed very subtle changes to be detected as a signal in proteomic and transcriptomic readouts without background noise that would be expected in an outpatient setting. In addition, as most immediate inflammatory reactogenicity to vaccines or adjuvants occurs within the first few days (see Figure 3), this setting allowed samples to be taken extremely frequently in the first 72 hours, to permit unique characterization of very early innate immune activation (see Figure 3), both at the transcriptomic and the proteomic level (acute phase proteins, cytokines, and chemokines).

### 3.2. The “Incarceration Effect”

Inpatient confinement marked a significant departure from previous applications of systems vaccinology. However, it introduces an unexpected but well recognized problem: the “*incarceration effect*” seen typically in variations in physiological parameters and safety laboratory readouts in healthy subjects who are confined for long periods, for example, in Phase 1 drug trials, where pharmacokinetics or specific restrictions mandate an inpatient regime with controlled diet and so forth. In such settings, changes in laboratory parameters may be observed within normal laboratory ranges and typically dismissed as “not clinically significant.” For example, in the BIOVACSAFE trials, a very distinct trend was seen in plasma proteins albumen and total protein with a gradual fall over time during the inpatient stay, followed by a marked jump between days 5 and 7 to a new set-point that was maintained at least 28 days. In contrast, acute phase proteins showed no baseline variation but were able to discriminate adjuvanted from nonadjuvanted vaccines in their response.

These observations raise an important question: if biomarkers are to provide useful alternatives to the standard clinical practice of identifying unacceptable levels of significant clinical reactogenicity (e.g., pain, fever, and redness measured in clinical trials) and identify subtle levels of reactogenicity that may only manifest a clinically significant problem late in clinical development or postmarketing, or in special groups (infants, pregnancy, and comorbidity), how will we distinguish these novel but subtle biomarkers from what has previously been dismissed as “not clinically significant”?

To do so will require a paradigm shift in thinking around preclinical toxicology and clinical pharmacovigilance and resultant regulatory guidance, especially for adjuvanted vaccines. As novel adjuvants are introduced, as personalized medicine becomes more common, and as vaccines are introduced for high-risk groups, these questions will have to be addressed if novel biomarkers of adjuvant reactogenicity are to shorten the cycle of discovery of unacceptable reactogenicity. The BIOVACSAFE program will set the limits of reactogenicity using safe licensed vaccines and adjuvants; further studies will have to employ more reactogenic or novel molecules that may manifest early or more frequent side effects.

### 3.3. Confirmation and Validation of Biomarkers Are Essential

**Figure 4:**
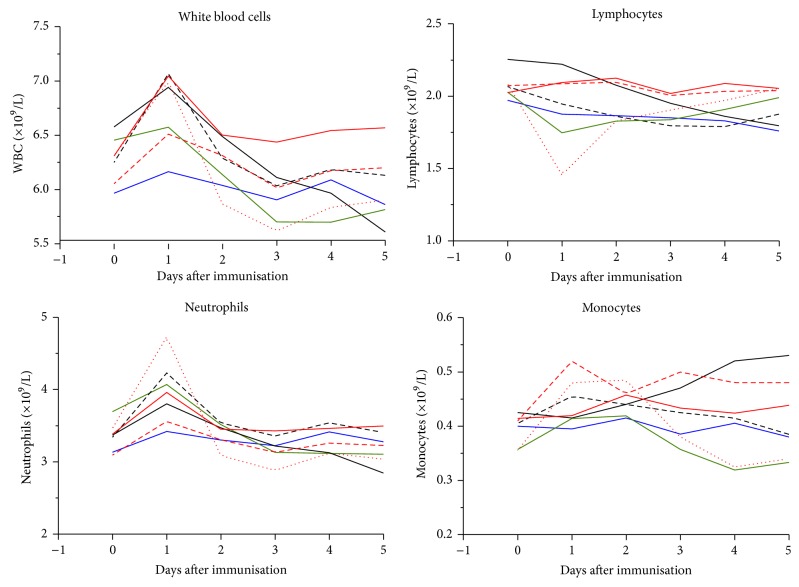
Differential kinetics of white blood cell populations enumerated by Coulter Counter at frequent time points following immunization with adjuvanted (red), unadjuvanted (green), or live (black) vaccines, or saline placebo.

With these considerations in mind follow-on clinical trials involving over 200 participants will be carried out by BIOVACSAFE, for example, to further characterize a biomarker profile identified with an adjuvanted influenza vaccine (Figure 2). A particular feature of this follow-on trial is that not only will whole blood be collected for RNA extraction and transcriptomics, but also cells will be separated into monocyte and granulocyte fractions. This results from initial observations in the inpatient trials that lymphocyte subpopulations have marked kinetics with differences between adjuvanted and nonadjuvanted as well as live vaccines (Figure 4), with peaks and troughs that are sometimes concordant and at other times discordant. Although a typical systems vaccinology study of adjuvant effect may separate peripheral blood mononuclear cells (PBMCs) in an attempt to control for cellular kinetics, without these pilot data characterizing the exact time course, the wrong time points may be selected. In addition, granulocyte populations are generally overlooked as standard gradient centrifugation preparations will not easily isolate these cells, while data from the BIOVACSAFE intensive inpatient trials highlights significant activation of neutrophils by adjuvanted vaccines (Figure 4). By careful observations of cellular kinetics following adjuvanted or nonadjuvanted immunization, the BIOVACSAFE project has guided the design of trials which can better address the innate/inflammatory axis and focus on population such as neutrophils that are central actors in innate responses.

### 3.4. Setting the Limits of Inflammatory Reactions to Vaccines and Adjuvants

A second problem with many systems vaccinology trials to date is that only nonreactogenic vaccines have generally been studied. To compensate for this, a large outpatient trial involving healthy adults having a booster immunization with a dTaP vaccine (diphtheria, tetanus, and acellular pertussis) has been organized to compare the transcriptomics, metabolomics, and proteomics profiles of subjects who may experience slightly more reactogenicity, within ethically acceptable parameters, largely as a result of diphtheria toxoid boosting. This will begin to explore the higher end of the scale of biomarkers of reactogenicity and act as validation for discriminatory biomarkers already observed.

### 3.5. Beyond the Clinical Trial: Validating Biomarkers against Natural Infections

One of the most significant features of a benefit-risk evaluation of a new vaccine or adjuvant is to contrast any adverse reactions from the immunization with those experienced during the infection that has been averted. The BIOVACSAFE consortium therefore includes pediatric cohorts in Ecuador and Germany: the former is collecting clinical samples from children being immunized with whole cell pertussis vaccines which are the standard EPI vaccines in Ecuador, to characterize the “omics” response to vaccines no longer routinely used in the EU; the latter is characterizing inflammatory responses in children presenting with natural infections. These data can act as a “positive” control to benchmark and place in context any subtle changes seen after immunization with licensed vaccines. By uniquely integrating data from transcriptomics and proteomics generated within a single core facility (Max Planck Institute for Infection Biology, Berlin) collected from adults and children undergoing immunization with adjuvanted and unadjuvanted vaccines or experiencing acute infection, the project is generating human data of unparalleled harmonization and direct comparability.

### 3.6. Beyond the Blood: Exploring Innate Immune Responses to Vaccine Adjuvants at the Site of Immunisation and Beyond

**Figure 5:**
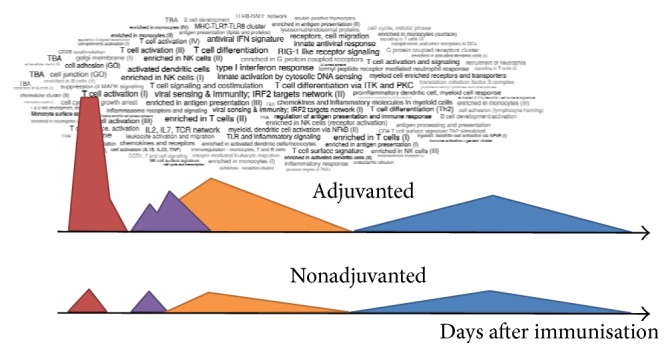
Cartoon illustrating relative expression of related genes over time for an adjuvanted and unadjuvanted vaccine, with the names of the associated overrepresented gene clusters in the first peak (red) for the adjuvanted vaccine identified in the cloud.

Integration and systems analysis of the trials is progressing, but initial analysis of whole blood transcriptomics changes from the intensive inpatient trials has already identified discriminatory transcriptomics profiles between adjuvanted and nonadjuvanted vaccines, with peaks at various times after immunisation in which pathways were active (*personal communication*, January Weiner, Max-Planck Institute For Infection Biology, Berlin, Figure 5). Interestingly, there was no obvious “incarceration effect” on transcriptomics patterns (in contrast to blood chemistry and some hematology parameters).

While systems vaccinology using whole blood or separated PBMCs has greatly advanced our understanding of the mechanisms of vaccine and adjuvant immunogenicity, events in the bloodstream are remote from the sites of immune activation such as the site of injection and draining lymph nodes. While these can be studied in animal models, there is an increased desire to undertake safe and ethical human immunology studies to generate data from humans. In a further refinement of the systems vaccinology model, BIOVACSAFE has embarked on a study in which a head-to-head comparison of adjuvanted vaccines for influenza (MF59C squalene microemulsion adjuvant) and hepatitis B sAg (ASO4C adjuvant, alum with TLR-agonist 3-*O*-desacyl-4′-monophosphoryl lipid A) is done, in which subjects will have a muscle biopsy at the site of injection, and from the contralateral leg as control, at different time points after immunisation: +3 hours and 1, 3, 5, and 7 days. RNA extraction and analysis of muscle transcriptomics will be compared with simultaneous whole blood. This is of particular relevance to adjuvanted vaccines as animal models have shown that while certain adjuvant systems (e.g., AS03, AS04) induce localised but also draining lymph node innate activation, associated with immunogenicity [32–34], others (e.g., MF59) induced a localized immunostimulatory environment in the muscle but did not modulate the transcriptome in the draining LN and do not induce any antigen-independent activation of B and T cells [35]. This unique direct comparison will enhance parallel studies in animal models including nonhuman primates.

The Bergström needle technique has been in use for many decades as a safe and effective way to obtain muscle including gene expression [36]. While techniques of fine needle aspiration of pathological or enlarged lymph nodes are also ethically possible, they give only a very limited amount of tissue from one site. BIOVACSAFE will take advantage of observations from the cancer literature that radiolabelled glucose Positron Emission Tomography (^18^F-FDG-PET) used in clinical practice to identify tissue with raised metabolism (glucose uptake) can image immune activation at both site of injection and draining lymph nodes in humans over the first 7 days after adjuvanted vaccines [37]. By combining a PET scan immediately prior to the muscle biopsy with characterization of muscle and blood transcriptomics profiles, BIOVASCAFE will characterize both the extent (distribution) and the intensity of draining lymph node activation after these two adjuvants and lay the foundation for future studies in which radiolabelled cytokine-specific ligands can be used to further dissect out immune responses at site of immunization with different adjuvants [38].

### 3.7. A New Language to Describe Adverse Events following Immunization (AEFIs) with Adjuvanted Vaccines for Systems Vaccinology

Another distinctive feature of the BIOVACSAFE collaboration is that all clinical and safety laboratory data have been harmonized to CDISC standards (http://www.cdisc.org/) at all clinical sites, making integration into centralized databases efficient and reliable for systems vaccinology analysis. This ensures that all data points are correctly and uniformly identified allowing portability and interoperability of data exchange across collaborators and external users and over time.

In order to systematically record and analyze AEFIs, various standard lexicons are used such as medDRA, developed by the International Conference on Harmonisation of Technical Requirements for Registration of Pharmaceuticals for Human Use (ICH) as “a rich and highly specific standardized medical terminology to facilitate sharing of regulatory information internationally for medical products used by humans” (http://www.meddra.org/). This takes a free text description of an adverse event by an investigator (e.g., pain, swelling, or inflammation at an injection site) and progressively translates it to a standardized term of wider scope. However, systems biology requires a standardized approach to data grouping that can be used to differentiate different participants, for example, “infected” or “uninfected.” Experience generated in the unique BIOVACSAFE clinical studies has revealed that it is difficult to select a single level of medDRA coding to direct the computer algorithms when comparing groups, as the level at which useful specificity may converge varies from event to event.

Table 1 illustrates the process for some AEFIs recorded during the BIOVACSAFE clinical trials of adjuvanted vaccines. As can be seen,* abdominal bloating*,* abdominal discomfort*, and* abdominal pain* remain split right up to the* higher level term*, leading to too few episodes in each to reach significance, when “discomfort” and “pain” are clearly closely related and could potentially be grouped during analysis. However, if integration takes place against the* higher level group*, they are combined with unrelated symptoms such as* nausea*. Similarly, as has been shown, significant changes in lymphocyte populations occur (Figure 4), and yet medDRA groups* increased* and* decreased neutrophil count* together with* any other white blood cell abnormality* at the* higher level term*. Furthermore, many linguistic synonyms such as “*reduced*” or “*low*” occur at the level of* preferred term* leading to possible splitting of related events at the clinical site, thereby reducing the power of the systems biology analysis. It is therefore extremely difficult, if not impossible, to direct the systems biology algorithms to a single preferred level in the medDRA hierarchy across all parameters. To compensate for this, significant postprocessing of AEFIs may be required to achieve a consensus level of precision, leading to potential bias or error. Finally, the* a priori* definition of reserved terms to apply only to immunization and not other study specific procedures such as phlebotomy may be required, to avoid mixing AEFIs with other procedures. In the BIOVACSAFE studies, the intensive inpatient trials were used to identify a lexicon of* preferred terms* most frequently observed that were incorporated in a drop-down menu on the electronic data capture forms to guide investigators to the most efficient way to classify AEFIs in follow-on trials. If systems biology is to be applied routinely in the clinical evaluation of adjuvanted vaccines, a major overhaul of how we report, classify, and grade AEFIs will be required, as is being pioneered in the BIOVACSAFE project.

### 3.8. Genetics of Adverse Reaction to Immunisation

As personalized medicine advances and adjuvanted vaccines are increasingly applied to populations that may be at risk of hyporesponsiveness or severe or autoimmune reactions, the genetic factors affecting immune responses may become important. This requires huge data sets at the population level to identify infrequent gene associations or adverse events such as autoimmunity. The involvement of deCODE in BIOVACSAFE, with access to the immunisation histories and clinical outcomes of thousands of Icelanders who have been genotyped and chip-typed, provides a powerful tool to answer such questions.

### 3.9. Animal Models of Reactogenicity to Immunization with Adjuvanted Vaccines

**Figure 6:**
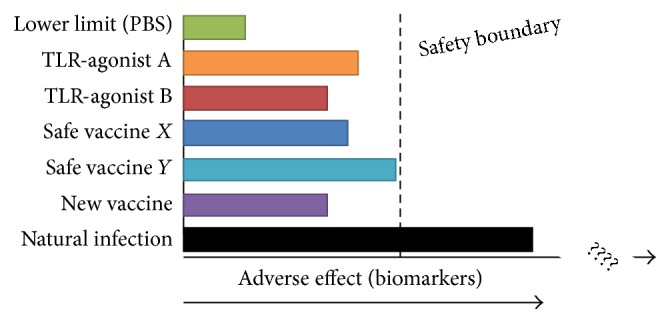
Illustration of need to identify a “safety boundary” on the sliding scale of adverse effects or biomarkers observed in response to “known to be safe” vaccines, immune agonists, and natural infection in animal models and human experimental medicine.

While a great deal can be achieved in human experimental medicine studies, and although it is increasingly accepted that animal models do not always reliably mimic the clinical experience, there are experiments that cannot ethically be conducted on humans; and rodents and rabbits remain the standard models for preclinical toxicology evaluation of adjuvanted vaccines. In addition, by studying only ethically acceptable and generally nonreactogenic vaccines in humans, it is difficult to know where the threshold of acceptability lies for any identified “biomarkers” (see Figure 6), in comparison, for example, with the inflammation induced by natural infection, which is assumed to be far greater than after immunisation. BIOVACSAFE will uniquely address this by integrating into the same data set human and animal data of transcriptomics and proteomics. Comparison will be made between human samples from the clinical trials and mice, rats, rabbits, and ferrets (blood, injection site, draining lymph nodes, spleen, liver, thymus, and bone marrow) immunized with the same adjuvanted and unadjuvanted vaccines or TLR-agonist positive controls, using a harmonized set of immune readouts (Table 2). Many bespoke assays have been created, particularly qPCR regents for ferret studies that are not commercially available, even though this species is an important influenza infection model. All these data will be integrated into a single integrated systems biology database for querying and analysis. Outputs may guide the use of appropriate preclinical toxicology models for novel adjuvants.

### 3.10. Putting It All Together: Integrated Database for Systems Vaccinology

**Figure 7:**
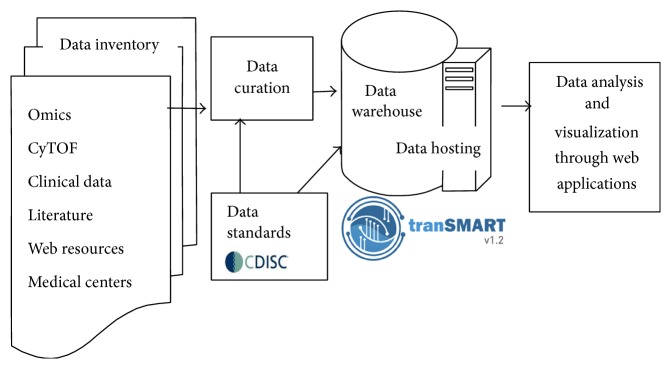
Integrated database for systems vaccinology.

Effective application of systems vaccinology requires the retrieval and integration of data from many different sites and assays, including preclinical and clinical data as well as complex laboratory and systems biology (or “omics”) data (Figure 7). BIOVACSAFE has developed a bespoke annotated large data warehouse using the open access tranSMART platform, including the provision of database hosting and curation as well as data mining capabilities. The use of the tranSMART platform allows the collection of data in a format that will be compatible with other international projects and consortia. Clinical data will meet CDISC-CDASH, CDISC-SDTM, and BRIDG UML standards to ensure seamless comparisons between trial protocols within BIOVACSAFE and externally or in the future. The shared database will enable partners to conduct exploration and analysis using a systems biology approach leading to biological interpretation, while preserving high standards of data protection and confidentiality. Data inventory requirements will be served by a standards compliant data repository that will store project data and metadata according to the list above. Once cleaned and curated, data will be accessed via a warehouse based on tranSMART for data mining and analytical processes. Data will then be accessible for export to specific systems biology and statistical tools for the analysis and correlation, after selection within the database on specific criteria. Statistical Analysis Plans will ensure that appropriate biological questions are framed in advance. This unique combination of adverse reactions, safety laboratory variables, and “omics” data from human and animal models will be an invaluable resource.

However, as with many fixed-term public-funded initiatives, a significant risk to the project remains the ongoing funding and availability of this resource after the project ends. It is to be hoped therefore that further public-private funding may invest in follow-on projects that capitalize on the information being generated using systems biology to investigate efficacy and reactogenicity of adjuvants and vaccines.

## Figures and Tables

**Table 1:** Examples of hierarchy of selected medDRA terms used to describe AEFIs in BIOVACSAFE clinical trials.

Preferred term	Higher level term	Higher level group
Abdominal distension	Flatulence bloating and distension	Gastrointestinal signs and symptoms
Abdominal discomfort	Gastrointestinal signs and symptoms not elsewhere coded	
Abdominal pain	Gastrointestinal and abdominal pains	
Nausea	Nausea and vomiting symptoms	

Neutrophil count decreased	White blood cell analyses		
Neutrophil count increased			

**Table 2:** Harmonized minimum data set of immune parameters for cross species evaluation of responses to adjuvanted vaccines and TLR agonists.

Vaccine or TLR agonist	Commercially available Luminex assays	Bespoke Luminex assays to mirror clinical studies	Bespoke qPCR plasmid calibrants for ferrets
Pentavalent/whole cell pertussis	IL-1*α*	C-reactive protein	IL1*β*
Trivalent influenza	IL-1*β*	Serum amyloid A	IL6
Trivalent influenza + MF59	IL-2	a2-macroglobulin	IL8
Engerix B	IL-3	LPS binding protein	CXCL10
Varicella vaccine	IL-4	Procalcitonin	CCL2
Poly I: C	IL-5	PTX3 pentraxin	IFN*α*
Lipopolysaccharide	IL-6	TREM-1	TNF*α*
Incomplete Freund's adjuvant	IL-9		Serum amyloid A
	IL-10		GAPDH
	IL-12 (p40)		RPL32
	IL-12 (p70)		
	IL-13		
	IL-17A		
	KC (CXCL1)		
	MCP-1 (CCL2)		
	MIP-1*α* (CCL3)		
	MIP-1*β* (CCL4)		
	RANTES		
	IFN-*γ*		
	TNF-*α*		
	GM-CSF		
	G-CSF		
	Eotaxin		
